# All-optical dynamic focusing of light via coherent absorption in a plasmonic metasurface

**DOI:** 10.1038/lsa.2017.157

**Published:** 2018-03-23

**Authors:** Maria Papaioannou, Eric Plum, Edward TF Rogers, Nikolay I Zheludev

**Affiliations:** 1 Optoelectronics Research Centre and Centre for Photonic Metamaterials, University of Southampton, Highfield, Southampton SO17 1BJ, UK; 2 Institute for Life Sciences, University of Southampton, Highfield, Southampton SO17 1BJ, UK; 3 Centre for Disruptive Photonic Technologies, School of Physical and Mathematical Sciences and The Photonics Institute, Nanyang Technological University, Singapore 637371, Singapore

**Keywords:** all-optical dynamic focusing, coherent control, coherent perfect absorption, metasurfaces

## Abstract

Vision, microscopy, imaging, optical data projection and storage all depend on focusing of light. Dynamic focusing is conventionally achieved with mechanically reconfigurable lenses, spatial light modulators or microfluidics. Here we demonstrate that dynamic control of focusing can be achieved through coherent interaction of optical waves on a thin beam splitter. We use a nanostructured plasmonic metasurface of subwavelength thickness as the beam splitter, allowing operation in the regimes of coherent absorption and coherent transparency. Focusing of light resulting from illumination of the plasmonic metasurface with a Fresnel zone pattern is controlled by another patterned beam projected on the same metasurface. By altering the control pattern, its phase, or its intensity, we switch the lens function on and off, and alter the focal spot’s depth, diameter and intensity. Switching occurs as fast as the control beam is modulated and therefore tens of gigahertz modulation bandwidth is possible with electro-optical modulators, which is orders of magnitude faster than conventional dynamic focusing technologies.

## Introduction

**Figure 1:**
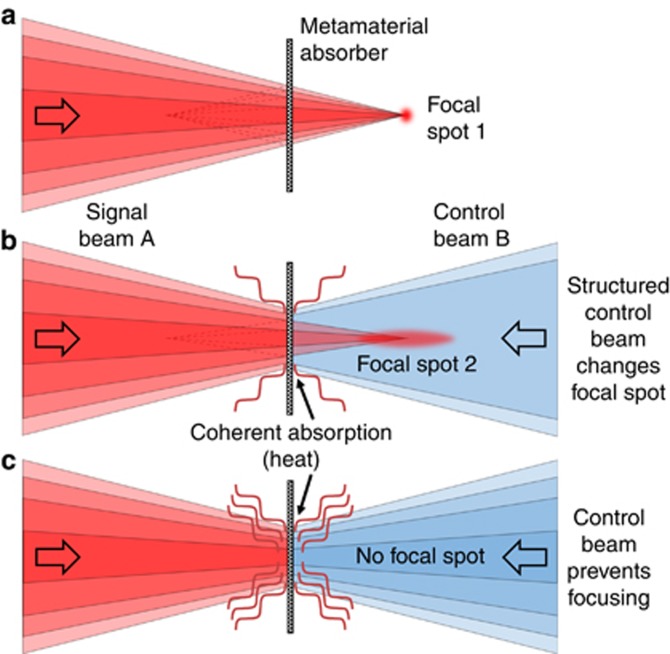
Dynamic optical focusing with a metasurface absorber of substantially sub-wavelength thickness. (**a**) A focused signal beam A produces focal spot 1. A coherent structured control beam B modifies signal beam A by controlling absorption on a metasurface absorber, producing (**b**) a modified focal spot 2 or (**c**) eliminating the focal spot altogether.

A light beam is focused by spatially varying its amplitude or phase distribution across the beam. Conventional convex lenses rely on the optical thickness of materials such as glass to introduce suitable phase delays, while Fresnel zone plates block light or introduce phase differences at certain distances from their centre in order to achieve constructive interference at the focus. Dynamic focusing is then realized by moving several solid lenses relative to each other^1^, by elastic deformation^2^, by varying curvature and optical thickness of microfluidic lenses^3, 4, 5, 6^, by reorientation of liquid crystals^7^ or by varying spatial intensity or phase profiles using spatial light modulators^8, 9, 10^. Such techniques are based on moving solid or liquid parts, or they rely on the reorientation of liquid crystal cells, making sub-millisecond response times difficult to achieve. While all-optical nonlinear self-focusing^11, 12, 13^ can be much faster, its inherent intensity dependence and minimum intensity requirements are rarely practical. In contrast, here we report dynamic control over optical focusing based on the linear interaction of light with light without moving parts, see Figure 1. Using coherent light, we image two Fresnel zone plate patterns onto opposite sides of a lossy metasurface beam splitter enabling dynamic control over intensity (‘on’ to ‘off’), depth (5 to 10 μm) and diameter (700 to 940 nm) of the focal spot as well as effective switching between a lens and an aperture. The focusing characteristics may be controlled continuously by modulating the phase of one illuminating beam. Tens of GHz modulation bandwidth can be achieved with telecommunications phase modulators, while the underlying light-matter interaction could deliver femtosecond-scale switching times^14, 15^.

The ability to control focusing arises from the fact that a sufficiently thin metasurface or beam splitter can be placed either at a node or anti-node of the standing wave formed by coherent counterpropagating beams of light. As the magnetic field of a normally incident plane wave cannot couple to a truly planar structure^16^, it is sufficient to consider the interaction between the wave’s electric field and the thin film. At a node, where the electric field is zero, light does not interact with the film, rendering it perfectly transparent. In contrast, at the anti-node the electric light-matter interaction will be maximized. In the case of an ideal lossy beam splitter absorbing 50% in a traveling wave^17^, this standing wave configuration allows continuous control of absorption from 0 to 100% by simply changing the relative position of the standing wave and the thin film^18^. (Such complete absorption is known as coherent perfect absorption and was first observed in optically thick media^19, 20, 21^.) On the contrary, in the case of an ideal lossless beam splitter (50:50), adjustment of the film’s position allows all illuminating light to be directed to one side of the beam splitter or the other. Coherent interaction of light with light on metasurface beam splitters of tens of nanometers thickness has been used to control intensity^18^, state of polarization^22^ and refraction^23^ of light at single photon intensities^24^ and with many THz bandwidth^14, 15^. Coherent absorption has also been observed in multi-layer graphene^25^ and a tunable graphene-based coherent absorber has been proposed^26^. Coherent control of light-metasurface interactions in standing waves has been applied to two-dimensional images for all-optical image processing^27^, image recognition^28^ and multi-channel logical data processing operations^29^. Here we demonstrate that coherent control can be useful in tasks requiring manipulation of light localization in three dimensions using the example of optical focusing. In essence, we show that coherent control of the interaction of light with a metasurface in a standing wave can effectively create a dynamically adjustable Fresnel zone plate.

## Materials and methods

### Metasurface fabrication and characterization

**Figure 2:**
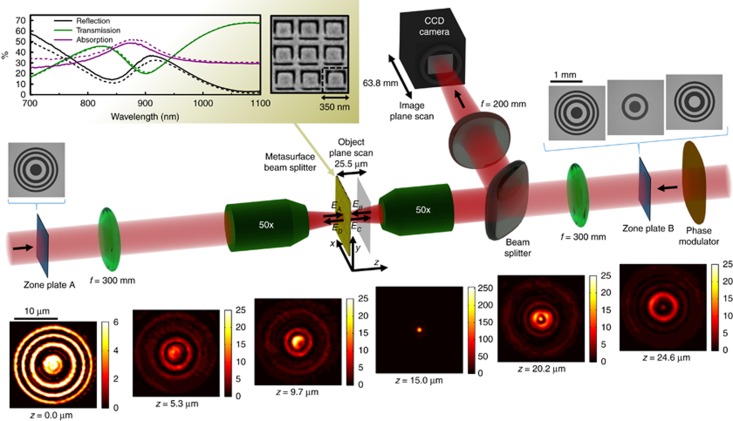
Coherent control of optical focusing is achieved by imaging Fresnel zone plates A and B onto opposite sides of a metasurface beam splitter using coherent light of 790 nm wavelength. At the metasurface, the images of the Fresnel zone plates A and B are demagnified 75 × by a combination of a 300-mm focal length lens and an infinity-corrected 4-mm focal length 50 × objective. Using the same objective combined with a 200-mm focal length lens, the field structure at different distances *z* from the metasurface is imaged onto a CCD camera by translating the camera along the optical path. The image sequence at the bottom shows intensity patterns recorded by the camera at different distances *z* when identical zone plates A and B are simultaneously imaged on either side of the lossy metasurface positioned near a node of the standing wave (*φ*=0) as in the third row of Figure 3. Here the intensity map taken at *z*=15 μm depicts the focal hotspot created by the Fresnel zone plates. The upper left inset shows a scanning electron micrograph of part of the metasurface alongside its traveling wave transmission, reflection and absorption spectra (where solid and dotted lines show measurements for opposite illumination directions). See Supplementary Fig. S1 for an enlarged unit cell with dimensions.

The metasurface beam splitter was manufactured by thermal evaporation of a 60-nm-thick gold layer on a 50-nm-thick silicon nitride membrane, subsequent silicon nitride removal by reactive ion etching to create a free-standing gold film and then focused ion beam milling of the aperture array shown by the scanning electron micrograph in Figure 2. The sample’s spectral response was measured with a microspectrophotometer, see upper left inset of Figure 2.

### Experimental characterization

**Figure 3:**
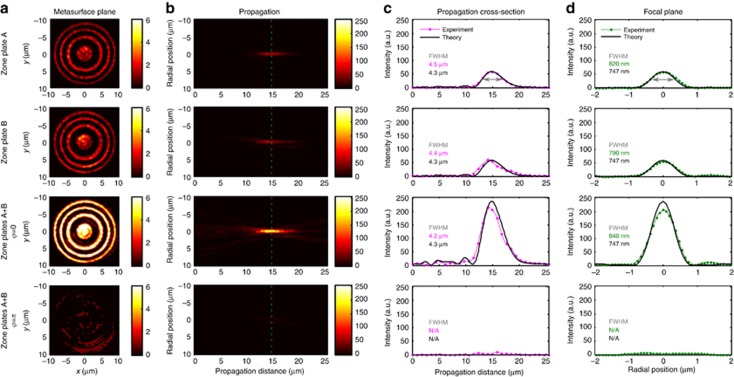
Controlling focal intensity by projecting identical Fresnel zone plate patterns, A (row 1) and B (row 2) onto opposite sides of the metasurface. Results in row 1 were measured by illuminating zone plate A while blocking beam B. Results in row 2 were measured by illuminating zone plate B while blocking beam A. Constructive interference of transmitted and reflected lens patterns leads to high focal intensity (*φ*=0, row 3), while destructive interference leads to vanishing focal intensity (*φ*=*π*, row 4). (**a**) Images of the metasurface plane taken with a camera when zone plate(s) are projected onto the metasurface. (**b**) Measured light distribution at different distances *z* from the metasurface (*yz*-plane) with the focal plane indicated by a dashed line. (**c**) Intensity distribution along the optical axis. (**d**) Radial intensity distribution on the focal plane at *z*=15 μm, obtained by averaging cross-sections along *x* and *y*. All color scales show intensity. See Supplementary Fig. S2 for more details.

Throughout this work, incident light is linearly polarized with the electric field parallel to the symmetry axis of the metasurface. We study coherent control of focusing of light with light for Fresnel zone plate lenses manufactured by photolithography from a 130-nm-thick chromium layer on a glass substrate, see Figure 2. Pairs of zone plates were imaged onto opposite sides of the metasurface with 75 × demagnification using light from the same 790-nm fiber-Bragg-grating-stabilized CW diode laser with 2-mW output power and less than 0.01-nm line width. Dynamic control over focusing was achieved by modulating the phase of light illuminating one zone plate with a liquid crystal phase modulator. Focusing of light resulting from transmission of zone plate image A and/or reflection of zone plate image B was characterized by imaging in front of the metasurface (object plane) starting at *z*=0 (metasurface plane) up to *z*=25.5 μm in steps of 880 nm with a CCD camera. Using an optical imaging system with 50 × magnification as shown, this was achieved by moving the camera 63.8 mm along the wave propagation direction in 2.2-mm steps, see the image sequence in Figure 2. The scanning length is well beyond the focal length, which was designed to be 15 μm in all cases. Similar intensity distributions are observed at equal *z*-distances on either side of the focal point, with differences caused by the inherently axially-asymmetric focusing of few-zone zone plates and deviations from rotational symmetry arising from experimental imperfections e.g. related to metasurface flatness and homogeneity. Maps showing the radial intensity distribution along the propagation direction (as in Figure 3b) were constructed from 30 images taken during a scan along the *z*-axis. The input beam intensities were chosen such that transmission of beam A and reflection of beam B have the same intensity when the zone plates are not present. The different experimental data sets studying (i) identical Fresnel zone plates, (ii) zone plates with a different number of rings and (iii) complementary zone plates use different camera exposure times to make optimal use of the camera’s dynamic range. Therefore, intensity is shown in the same arbitrary units within each data set, but different data sets use different arbitrary units.

### Numerical characterization

Theoretical light distributions were calculated based on the angular spectrum method^30, 31^. To aid comparison between experimental and theoretical intensity distributions, the arbitrary intensity units of each of the three theoretical data sets have been chosen such that the theoretical focal peak intensity of Fresnel zone plate A matches that of the corresponding experimental data set. A normalized intensity scale was used in the last figure, as explained in its caption.

### Characterization of focal spots

The focal diameter was determined as the full width at half maximum (FWHM) of the focal spot intensity in the *xy*-plane at *z*=15 μm. The focal depth was determined as the FWHM of the focal spot intensity along the optical axis (*z*-axis).

## Results and discussion

Following Ref. 32, the interaction of incident counterpropagating coherent light fields, *E*_*A*_(*x*, *y*) and *E*_*B*_(*x*, *y*), on a thin film with complex field transmission and reflection coefficients, *t* and *r*, may be described by the resulting output fields *E*_*C*_=*tE*_*A*_+*rE*_*B*_ and *E*_*D*_=*rE*_*A*_+*tE*_*B*_, where *E*_*i*_=*E*_*i*_(*x*, *y*) are the input and output field distributions immediately before and after interaction with the thin film. When considering the local interaction of the field with the thin film, it is convenient to consider the phase difference between the incident fields *θ*=arg(*E*_*B*_)−arg(*E*_*A*_), while the output beams may be conveniently described in terms of the phase difference between transmission and reflection of different input beams *φ*=arg(*rE*_*B*_)−arg(*tE*_*A*_). These phases are related by *φ*=*θ*+arg(*r*)−arg(*t*).

Let us consider the simplest case of imaging identical Fresnel zone plates on opposite sides of the beam splitter. For any beam splitter, we can choose the incident intensities such that transmission of field A and reflection of field B only differ by the phase *φ*, which corresponds to *rE*_*B*_=*tE*_*A*_*e*^*iφ*^. In this case, *E*_*C*_=*tE*_*A*_(1+*e*^*iφ*^), implying that the phase of light field B allows control over output field C, from 4-fold intensity enhancement for *φ*=0 (intensity ∝|*E*|^2^) to complete suppression for *φ*=*π*. If the beam splitter is planar, then *t*=*r*+1 and destructive interference of incident fields corresponding to *E*_*B*_=−*E*_*A*_ renders it perfectly transparent resulting in *E*_*C*_=*E*_*A*_ and *E*_*D*_=*E*_*B*_.

It is instructive to consider the limiting cases of ideal, planar lossy and lossless beam splitters. The limiting case of an ideal lossy beam splitter corresponds to *r*=−0.5. It follows that *φ*=*θ*+*π* and *E*_*D*_=−*E*_*C*_, implying identical focusing behaviour in both directions. When identical Fresnel zone plates are imaged on the ideal lossy beam splitter, constructive interference of the incident fields (*θ*=0) results in coherent perfect absorption of all light, *E*_*C*_=*E*_*D*_=0 (as in Figure 1c), while destructive interference on the beam splitter (*θ*=*π*) results in coherent perfect transparency and maximum intensity of both output fields.

The other limiting case is an ideal lossless beam splitter that is described by 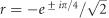, implying *φ*=*θ*±*π*/2 and interchanging output fields *E*_*C*_ and *E*_*D*_ for a *π* change in *θ*. When identical zone plates are imaged on the ideal lossless beam splitter with equal intensity, phase differences 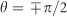 of the incident fields result in focusing of all incident light into one focus on one side of the beam splitter, 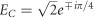 and *E*_*D*_=0 or vice versa.

Here we consider a lossy metasurface beam splitter that absorbs about 34% of a single illuminating beam at the experimental wavelength of 790 nm, placing it closer to the ideal lossy beam splitter considered above than to the lossless one (see spectral response in Figure 2). The metasurface beam splitter consists of a 60-nm-thick free-standing gold film perforated with an array of asymmetrically split ring apertures with 350-nm period and an overall size of 100 μm × 100 μm. This nanostructure, also known as planar metamaterial or metasurface, has almost identical properties for illumination of opposite sides and its level of absorption is controlled by the split ring aperture dimensions^33^.

We study coherent control of focusing of light with light for Fresnel zone plate lenses as explained in the Materials and Methods section and illustrated by Figure 2. Pairs of zone plates were imaged onto opposite sides of the metasurface using light from the same laser and dynamic control over focusing was achieved by modulating the phase of light illuminating one zone plate. Focusing of light was characterized by imaging the intensity distribution from the metasurface at *z*=0 to a distance of *z*=25.5 μm in front of the metasurface, which is well beyond the focal length, that was designed to be *z*=15 μm in all cases.

### Controlling focal intensity with light

Figure 3 illustrates coherent control of focal intensity with light, which results from imaging of identical Fresnel zone plates onto opposite sides of the metasurface. The projections of the individual zone plates onto the metasurface and the resulting focusing characteristics are shown by the first two rows. Both zone plates form similar focal spots with about 15-μm focal length, 4.4-μm focal depth and 800-nm focal diameter. Simultaneous projection of both lens patterns onto the metasurface does not affect the focal depth and diameter within experimental accuracy, however, the focal intensity becomes strongly dependent on the phase difference of the illuminating beams. Constructive interference of transmission of lens pattern A and reflection of lens pattern B (*φ*=0, row 3) leads to a 4-fold intensity increase, while destructive interference (*φ*=*π*, row 4) results in almost complete absence of the focal spot. While our simulations predict that the focal intensity should reduce to zero for *φ*=*π*, we observe a residual intensity of about 7% of the peak intensity in our experiments, corresponding to 1400% contrast between maximum and minimum focal intensity. The residual intensity arises from experimental imperfections related to the accuracy of phase control and alignment as well as metasurface flatness and homogeneity. The focal intensity can be controlled continuously between these extremes by varying *φ* from 0 to *π*.

### Controlling focal depth and diameter

**Figure 4:**
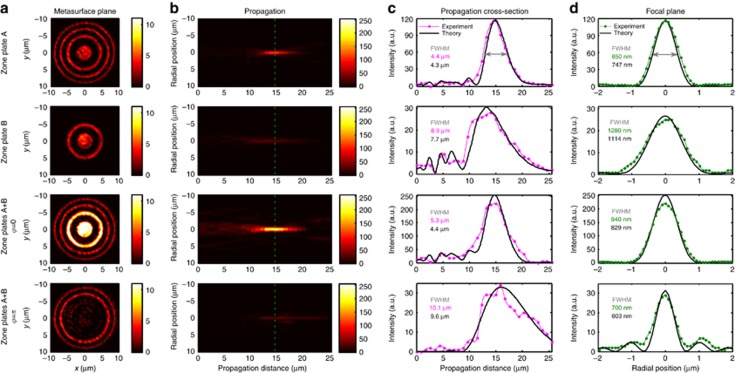
Controlling focal depth and diameter by projecting different Fresnel zone plate patterns, A (row 1) and B (row 2) onto opposite sides of the metasurface. Results in rows 1 and 2 were recorded by illuminating only one zone plate (as in Figure 3). Constructive interference of transmitted and reflected lens patterns leads to a short, high-intensity focus (*φ*=0, row 3), while destructive interference leads to a dimmer, narrower Bessel-beam-like focus with a large focal depth (*φ*=*π*, row 4). (**a**) Images of the metasurface plane taken with a camera when zone plate(s) are projected onto the metasurface. (**b**) Measured light distribution at different distances *z* from the metasurface (*yz*-plane) with the focal plane indicated by a dashed line. (**c**) Intensity distribution along the optical axis. (**d**) Radial intensity distribution on the focal plane at *z*=15 μm, obtained by averaging cross-sections along *x* and *y*. All color scales show intensity. See Supplementary Fig. S3 for more details.

Figure 4 illustrates coherent control of focal depth and diameter with light, which results from imaging Fresnel zone plates with a different number of rings onto opposite sides of the metasurface, where the smaller zone plate corresponds to the central part of the larger one. The projections of the individual zone plates onto the metasurface and the resulting focusing characteristics are shown by the first two rows. The smaller 2-ring zone plate has an increased focal depth and diameter compared to the 4-ring zone plate. Simultaneous projection of both lens patterns onto the metasurface creates an effective zone plate, where the phase *φ* controls the contribution of the inner rings from 4-fold intensity enhancement (*φ*=0, row 3 of Figure 4a) to complete suppression (*φ*=*π*, row 4 of Figure 4a) compared to the outer rings.

Enhancement of the inner rings yields similar focusing characteristics to the original 4-ring zone plate. In contrast, suppression of the inner rings yields a focus resembling an optical needle^34^ or a Bessel beam^35, 36^, which has a very long focal depth of 10 μm and a small, sub-wavelength focal diameter of only 700 nm. Thus, variation of *φ* from 0 to *π* allows the focal depth to be controlled from 5–10 μm and the focal diameter that determines the resolution to be controlled from 700–940 nm, corresponding to dynamic ranges of 100% and 35%, respectively.

### Turning a lens into a perfect absorber or an aperture

**Figure 5:**
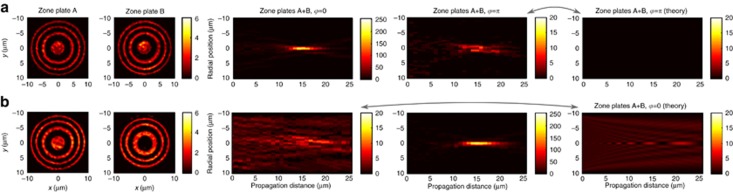
From focusing zone plate to diffracting aperture or darkness. Interaction of (**a**) identical and (**b**) inverted Fresnel zone plate patterns A and B on the metasurface beam splitter. When the transmitted lens pattern A and the reflected lens pattern B are in phase (*φ*=0), identical zone plates yield a bright focal spot, while inverse zone plates add up to a diffracting circular aperture. When the zone plate patterns are out-of-phase (*φ*=*π*), identical zone plates block all light, while inverted zone plates focus. For clarity, measured and simulated light propagation are shown on a reduced intensity scale for the effective aperture and absorber cases. Column 1 shows the projection of zone plate A onto the metasurface at *z*=0 when blocking beam path B. Column 2 shows the projection of zone plate B while blocking path A. For simultaneous illumination of both zone plates, columns 3 and 4 show measured and column 5 shows simulated intensity distributions for different propagation distances *z*. All color scales show intensity. See Supplementary Figs. S4 and S5 for detailed results on inverted zone plate patterns.

As illustrated by Figure 3, projection of identical zone plate patterns onto the metasurface allows control over the focal intensity in a way that corresponds to gradually interchanging the lens with a perfect absorber. This changes the intensity of the focal spot from ‘on’ to ‘off’ without affecting the structure of the spatial light distribution. While the intensity in the ‘off’ state (*φ*=*π*) is predicted to be zero, we observe a residual focal intensity of a few percent (as explained above) that is shown on Figure 5a using different color scales for high and low intensity light distributions. Instead of blocking (almost) all light, focal intensity may also be controlled by effectively removing the lens. This is achieved by projecting inverted zone plate patterns onto the metasurface as illustrated by Figure 5b. Depending on the phase difference between the projected inverse zone plate patterns, their superposition on the metasurface forms a homogeneously illuminated circular disk (*φ*=0) or rings of equal intensity but opposite phase (*φ*=*π*). Thus, the inverted zone plate patterns for *φ*=*π* effectively create a phase Fresnel zone plate on the metasurface that produces essentially the same focal spot as identical zone plate patterns create for *φ*=0 (compare Figure 5b column 4 with Figure 5a column 3), while the homogeneous circular disk generated by inverted zone plate patterns for *φ*=0 behaves like a lens-sized aperture illuminated by a plane wave. Numerical modelling clearly reveals the aperture diffraction pattern and its main features are also observed experimentally, compare Figure 5b columns 3 and 5.

### Phase-dependence of focusing properties

**Figure 6:**
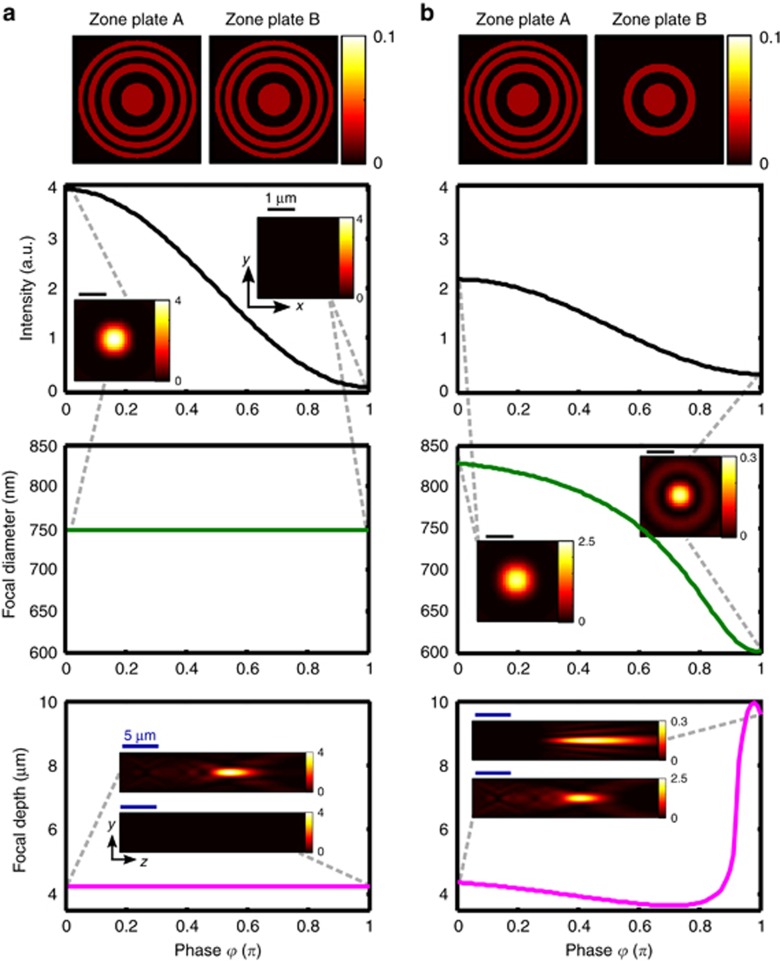
Simulated phase-dependence of focal properties for interaction of (**a**) identical Fresnel zone plate patterns (as in Figure 3) and (**b**) Fresnel zone plate patterns of different size (as in Figure 4) on a beam splitter. The intensity is normalized by the focal intensity produced by lens pattern A alone. Row 1 shows the intensity distribution for each zone plate pattern alone, rows 2 and 3 show the peak intensity and FWHM focal diameter at the nominal focal distance of *z*=15 μm. Row 4 shows the FWHM focal depth. All color scales show intensity.

Throughout all experiments, our experimental results closely match theoretical predictions based on the angular spectrum method^30, 31^. Figure 6 shows the modelled phase-dependence of focal intensity, depth and diameter for identical zone plates (as in Figure 3) and for zone plates with a different number of rings (as in Figure 4), illustrating that phase modulation can indeed provide continuous control over all focusing characteristics between the experimentally observed extremes.

## Conclusions

Although we use Fresnel zone plate patterns written in metal films, it does not matter how the light fields that are imaged onto the metasurface beam splitter are created. Conventional lenses, lens arrays, gratings, holograms or other optical elements may be imaged onto the beam splitter instead. Therefore, our approach may be applied to achieve fast modulation of various optical functionalities. By imaging the beam splitter itself onto a different plane—such as the camera in our experiments—the dynamic focusing or other functionality can also be applied outside of the interferometer. While this is beyond the scope of our experimental study, we note that our approach can be extended to manipulation of other focal characteristics such as the focal length (see Supplementary Information and Supplementary Fig. S6) and optimized to achieve control over one focal property while minimizing the effect on other focal properties through zone plate design.

In summary, we demonstrate dynamic control over optical focusing by projecting Fresnel zone plate patterns onto opposite sides of a metasurface beam splitter using coherent light. We control focal intensity from ‘on’ to ‘off’, focal depth with a dynamic range of 100%, focal diameter by 35% and effectively replace a lens by an aperture without moving parts. Instead, focusing is controlled by an optical phase modulator, implying that modulation rates of tens of GHz are accessible using telecommunications phase modulators.
