# Supplementary material for: All-optical dynamic focusing of light via coherent absorption in a plasmonic metasurface
Source: Light Sci Appl. 2018 Mar 23;7:17157–. doi: 10.1038/lsa.2017.157 (PMC6060048; doi:10.1038/lsa.2017.157)
Supplement: Supplementary Information [file lsa2017157x1.pdf]

Supplementary information for
"All-optical dynamic focusing of light via coherent absorption in a plasmonic metasurface"

Maria Papaioannou[1], Eric Plum[1, *], Edward T. F. Rogers[1, 2], Nikolay I. Zheludev[1, 3, †]

[1]Optoelectronics Research Centre and Centre for Photonic Metamaterials,
University of Southampton, Highfield, Southampton, SO17 1BJ, UK
[2]Institute for Life Sciences, University of Southampton, Highfield, Southampton, SO17 1BJ, UK
[3]Centre for Disruptive Photonic Technologies, School of Physical and Mathematical Sciences and
The Photonics Institute, Nanyang Technological University, Singapore 637371
* erp@orc.soton.ac.uk † niz@orc.soton.ac.uk URL: www.nanophotonics.org.uk

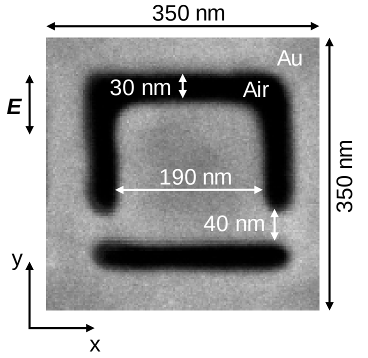

Figure S1: Unit cell of the fabricated metasurface. Scanning electron micrograph of a single metasurface unit cell with dimensions. The gold layer is 60 nm thick and the overall size of the metasurface array is 100 µm × 100 µm. The electric field direction *E* of incident electromagnetic waves is indicated.

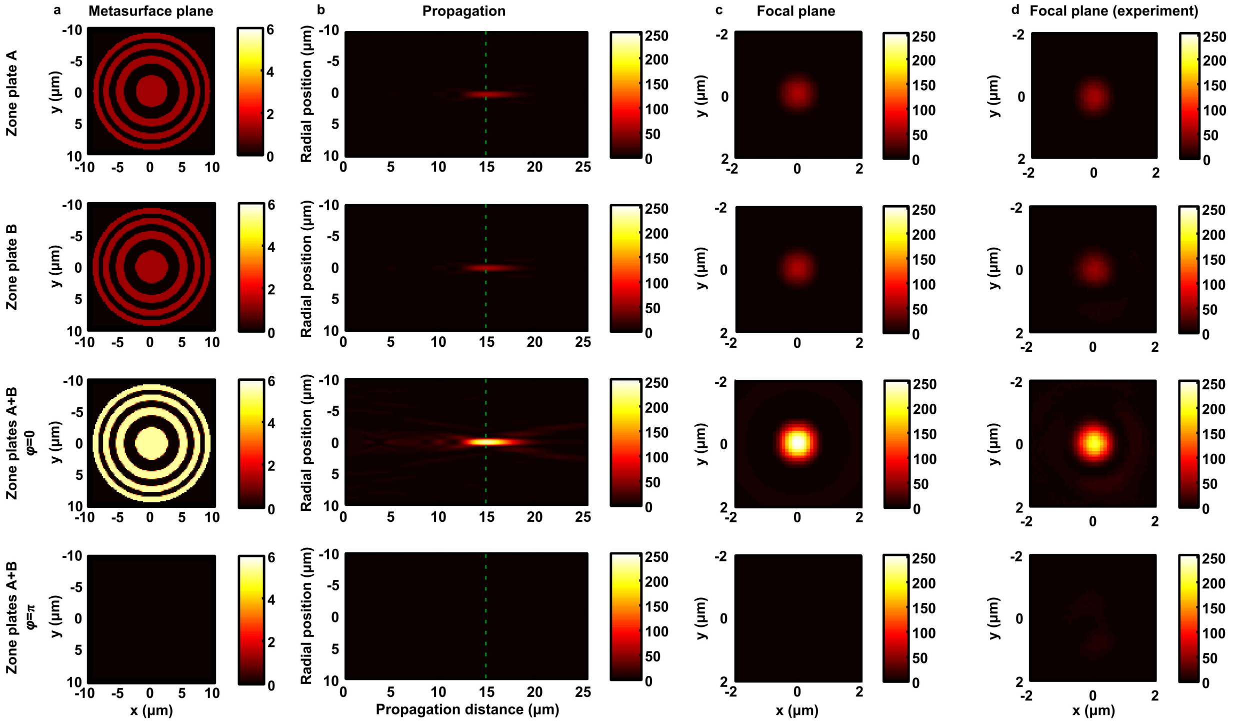

Figure S2: More on controlling focal intensity by projecting identical Fresnel zone plate patterns, A (row 1) and B (row 2) onto opposite sides of the metasurface. Results in row 1 correspond to illumination of zone plate A while blocking beam B. Results in row 2 correspond to illumination of zone plate B while blocking beam A. Constructive interference of transmitted and reflected lens patterns leads to high focal intensity ($\varphi = 0$, row 3), while destructive interference leads to vanishing focal intensity ($\varphi = \pi$, row 4). (**a**) Simulated intensity on the metasurface plane. (**b**) Simulated light distribution at different distances *z* from the metasurface (*yz* -plane) with the focal plane indicated by a dashed line. (**c**) Simulated intensity distribution on the focal plane at $z = 15$ µm. (**d**) Measured intensity distribution on the focal plane at $z = 15$ µm. All color scales show intensity.

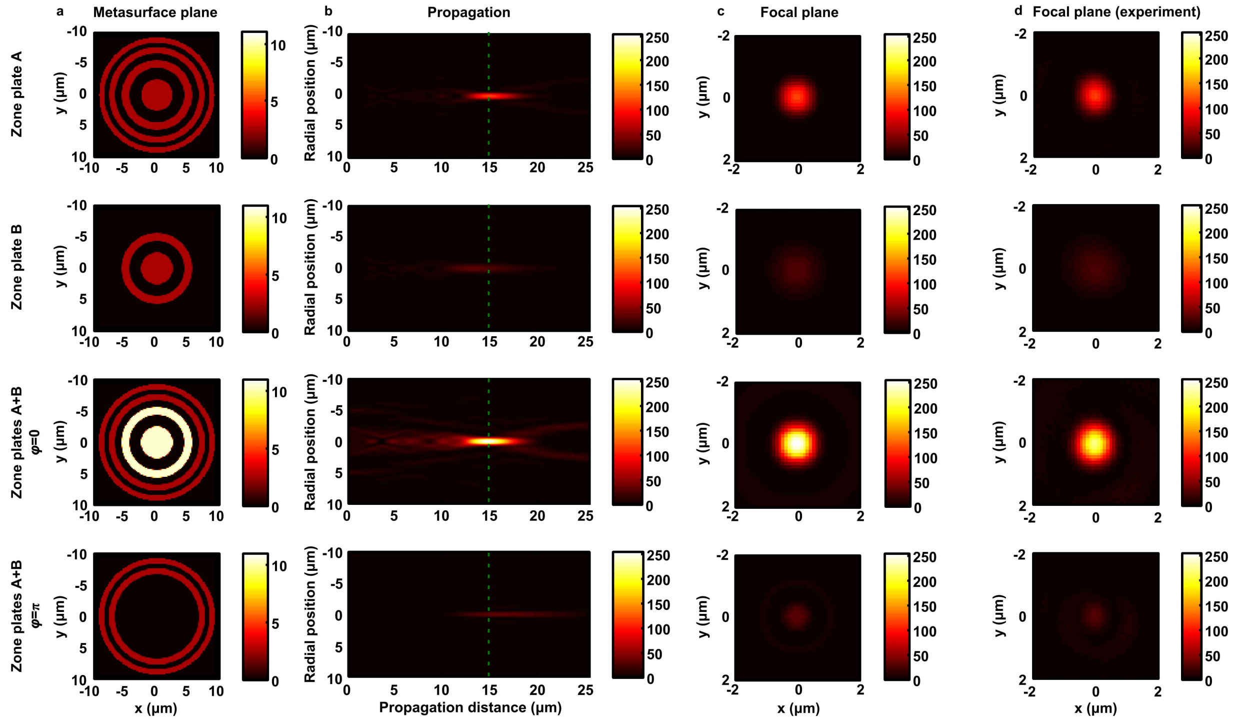

Figure S3: More on controlling focal depth and diameter by projecting different Fresnel zone plate patterns, A (row 1) and B (row 2) onto opposite sides of the metasurface. Results in row 1 correspond to illumination of zone plate A while blocking beam B. Results in row 2 correspond to illumination of zone plate B while blocking beam A. Constructive interference of transmitted and reflected lens patterns leads to a short, high-intensity focus ($\varphi = 0$, row 3), while destructive interference leads to a dimmer, narrower Bessel-beam-like focus with a large focal depth ($\varphi = \pi$, row 4). (**a**) Simulated intensity on the metasurface plane. (**b**) Simulated light distribution at different distances z from the metasurface (yz -plane) with the focal plane indicated by a dashed line. (**c**) Simulated intensity distribution on the focal plane at z = 15 μm. (**d**) Measured intensity distribution on the focal plane at z = 15 μm. All color scales show intensity.

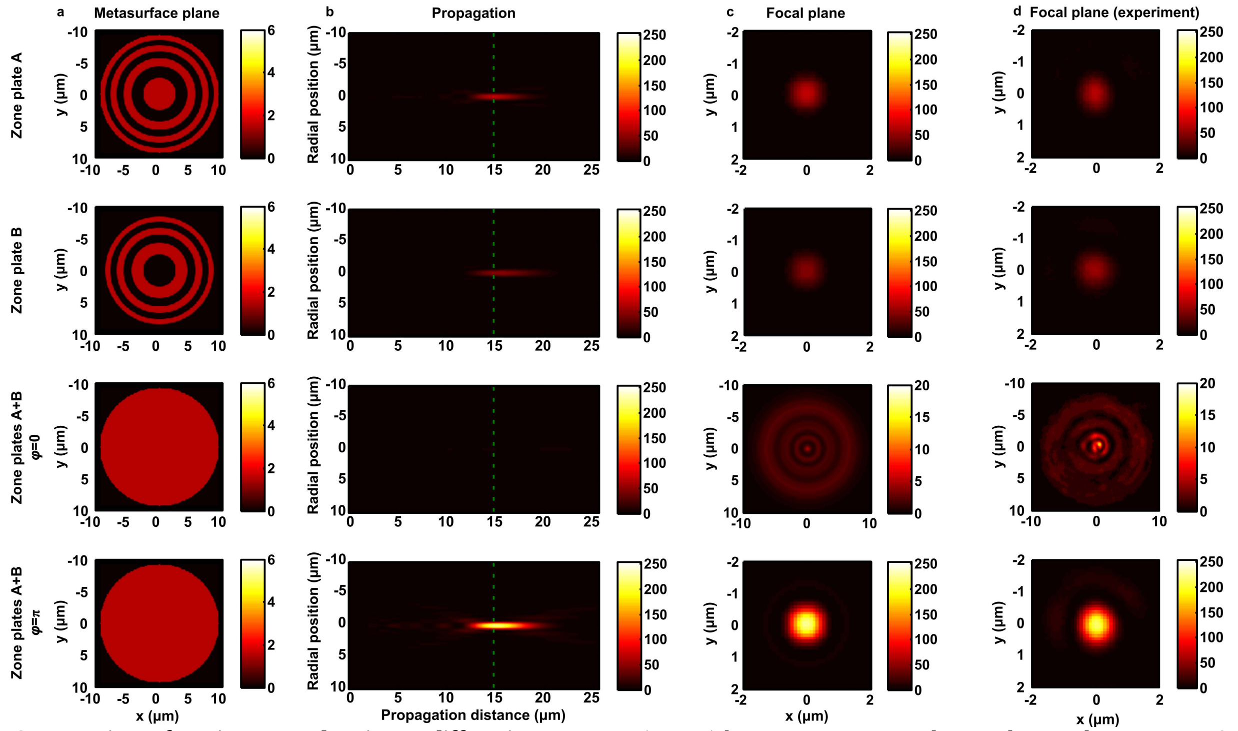

Figure S4: Turning a focusing zone plate into a diffracting aperture (**part 1**) by projecting inverted Fresnel zone plate patterns, A (row 1) and B (row 2) onto opposite sides of the metasurface. Results in row 1 correspond to illumination of zone plate A while blocking beam B. Results in row 2 correspond to illumination of zone plate B while blocking beam A. When the transmitted and reflected lens patterns are in phase, they combine to form an effective aperture ($\varphi = 0$, row 3), while an effective phase zone plate with a high intensity focal spot results from a π phase difference of these fields ($\varphi = \pi$, row 4). (**a**) Simulated intensity on the metasurface plane. (**b**) Simulated light distribution at different distances z from the metasurface (yz -plane) with the focal plane indicated by a dashed line. (**c**) Simulated intensity distribution on the focal plane at z = 15 μm. (**d**) Measured intensity distribution on the focal plane at z = 15 μm. All color scales show intensity.

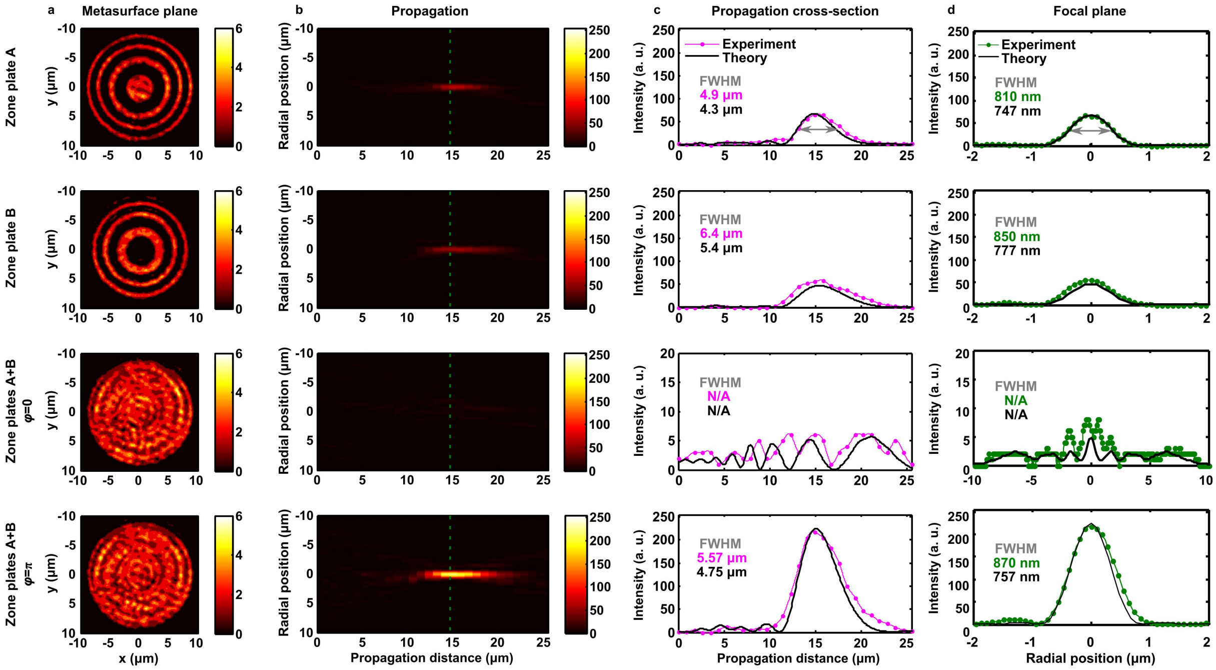

Figure S5: Turning a focusing zone plate into a diffracting aperture (**part 2**) by projecting inverted Fresnel zone plate patterns, A (row 1) and B (row 2) onto opposite sides of the metasurface. Results in row 1 were measured by illuminating zone plate A while blocking beam B. Results in row 2 were measured by illuminating zone plate B while blocking beam A. When the transmitted and reflected lens patterns are in phase, they combine to form an effective aperture ($\varphi = 0$, row 3), while an effective phase zone plate with a high intensity focal spot results from a π phase difference of these fields ($\varphi = \pi$, row 4). (**a**) Images of the metasurface plane taken with a camera when zone plate(s) are projected onto the metasurface. (**b**) Measured light distribution at different distances z from the metasurface (yz -plane) with the focal plane indicated by a dashed line. (**c**) Intensity distribution along the optical axis. (**d**) Radial intensity distribution on the focal plane at z = 15 µm, obtained by averaging cross-sections along x and y. All color scales show intensity.

Controlling focal length

Here we present a numerical example of how coherent absorption in a plasmonic metasurface also allows the focal length of optical focusing to be controlled with light, see Figure S6. For consistency, we consider the same zone plate A as in the main manuscript (row 1 of panel a). Zone plate B (row 2 of panel a) partially overlaps with zone plate A such that coherent interaction of both patterns alters the central zone radius as well as the width and total number of rings that contribute to focusing (rows 3 and 4 of panel a), resulting in focal spots at different distances for different optical phase differences φ (rows 3 and 4 of panels b and c). The zone radii of zone plate B were chosen by varying the radii of the design of zone plate A aiming to create two distinct focal points corresponding to constructive (φ=0) and destructive (φ=π) interference of the transmitted and reflected lens patterns. Zone plates A and B create similar focal spots around the initial design focal distance of z=15 µm (rows 1 and 2). Their coherent interaction on the metasurface results in a focal spot at z=15 µm for φ=0 and at z=10.3 µm for φ=π. This translation of the focal distance by Δf=4.7 µm corresponds to a 31% reduction of the original focal distance f. We note that, in this example, the focal spot at z=10.3 µm when φ=π has 96 nm wider focal diameter, 1.4 µm longer focal depth and about 5% intensity compared to the focal spot created at z=15 µm when φ=0. The difference in focal intensities could be compensated by modulating the laser intensity or avoided by designing *both* zone patterns specifically for dynamic control of the focal length.

To optimise focal length switching, both zone plates could be designed to create a pair of equal intensity focal spots, at the same distances f_1 and f_2. If these zone plates are designed such that they create the spots at f_1 with the same phase, but the spots at f_2 with opposite phases, then phase modulation could enhance the focal spot at f_1 while suppressing the focal spot at f_2 and vice versa. Suitable zone plates would be complex, but they could be generated by grayscale superposition of conventional Fresnel zone plate patterns or by superposition of conventional "transparent and opaque" Fresnel zone plate patterns with reduced width of the transparent rings (in order to make space for the second pattern). While design is non-trivial, custom plates have been previously designed to satisfy similarly complex requirements[1-3].

Thus, zone plate patterns can be designed for all-optical control of focal intensity, focal depth, focal diameter and focal distance.

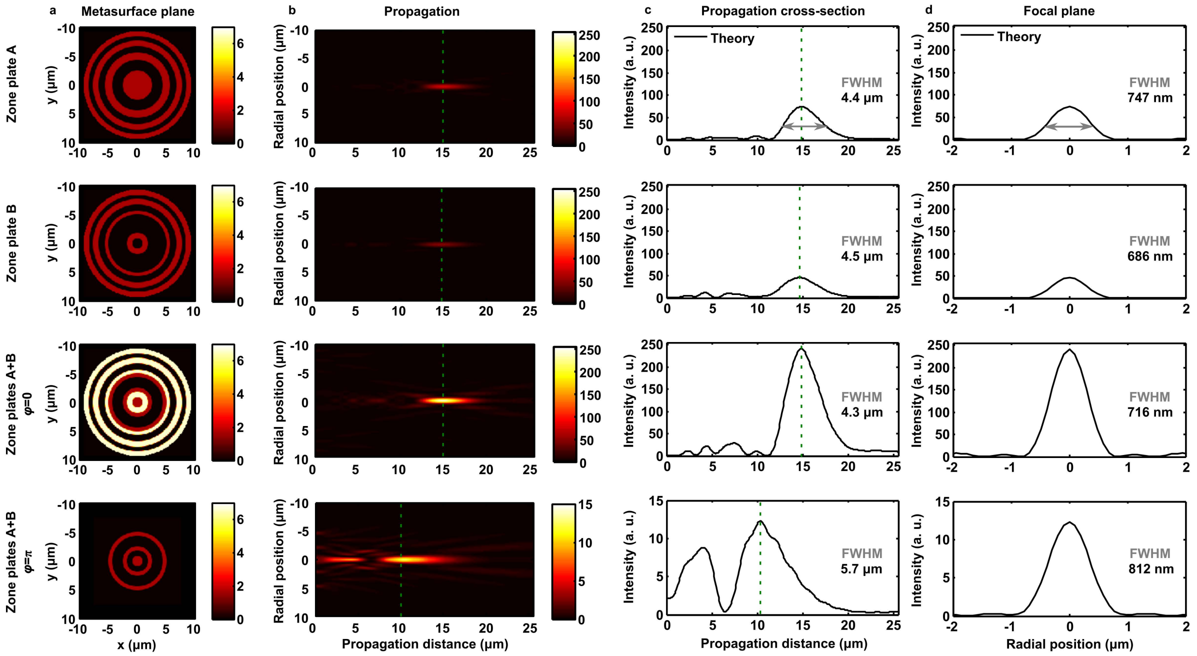

Figure S6: Controlling the focal distance by projecting Fresnel zone plate patterns, A (row 1) and B (row 2) consisting of a combination of identical and partially overlapping zones onto opposite sides of the metasurface. Results in row 1 correspond to illumination of zone plate A while blocking beam B. Results in row 2 correspond to illumination of zone plate B while blocking beam A. When the transmitted and reflected lens patterns are in phase, they combine to form a focal spot at z = 15 µm ($\varphi = 0$, row 3), while a focal spot at z = 10.3 µm results from a π phase difference of these fields ($\varphi = \pi$, row 4). (**a**) Simulated intensity on the metasurface plane. (**b**) Simulated light distribution at different distances z from the metasurface (*yz*-plane) with the focal plane indicated by a dashed line for each case. (**c**) Simulated intensity distribution along the optical axis. (**d**) Simulated radial intensity distribution on the focal plane, indicated by the dashed line in (**b**) for each case. All color scales show intensity.

1 Rogers ETF, Lindberg J, Roy T, Savo S, Chad JE *et al.*, A super-oscillatory lens optical microscope for subwavelength imaging. *Nat Mater* 2012; **11**: 432.

2 Yuan G, Rogers ETF, Roy T, Adamo G, Shen Z *et al*. Planar super-oscillatory lens for sub-diffraction optical needles at violet wavelengths. *Sci Rep* 2014; **4**: 6333.

3 Yuan GH, Rogers ETF, Zheludev NI. Achromatic super-oscillatory lenses with sub-wavelength focusing. *Light Sci Appl* 2017; **6**: e17036.
